# Combining Multi-Dimensional Convolutional Neural Network (CNN) With Visualization Method for Detection of *Aphis gossypii* Glover Infection in Cotton Leaves Using Hyperspectral Imaging

**DOI:** 10.3389/fpls.2021.604510

**Published:** 2021-02-15

**Authors:** Tianying Yan, Wei Xu, Jiao Lin, Long Duan, Pan Gao, Chu Zhang, Xin Lv

**Affiliations:** ^1^College of Information Science and Technology, Shihezi University, Shihezi, China; ^2^Key Laboratory of Oasis Ecology Agriculture, Shihezi University, Shihezi, China; ^3^College of Agriculture, Shihezi University, Shihezi, China; ^4^Xinjiang Production and Construction Corps Key Laboratory of Special Fruits and Vegetables Cultivation Physiology and Germplasm Resources Utilization, Shihezi, China; ^5^College of Biosystems Engineering and Food Science, Zhejiang University, Hangzhou, China; ^6^Key Laboratory of Spectroscopy Sensing, Ministry of Agriculture and Rural Affairs, Hangzhou, China; ^7^School of Information Engineering, Huzhou University, Huzhou, China

**Keywords:** *Aphis gossypii* Glover, machine learning, aphid infection, hyperspectral imaging, convolutional neural network (CNN), visualization

## Abstract

Cotton is a significant economic crop. It is vulnerable to aphids (*Aphis gossypii* Glovers) during the growth period. Rapid and early detection has become an important means to deal with aphids in cotton. In this study, the visible/near-infrared (Vis/NIR) hyperspectral imaging system (376–1044 nm) and machine learning methods were used to identify aphid infection in cotton leaves. Both tall and short cotton plants (Lumianyan 24) were inoculated with aphids, and the corresponding plants without aphids were used as control. The hyperspectral images (HSIs) were acquired five times at an interval of 5 days. The healthy and infected leaves were used to establish the datasets, with each leaf as a sample. The spectra and RGB images of each cotton leaf were extracted from the hyperspectral images for one-dimensional (1D) and two-dimensional (2D) analysis. The hyperspectral images of each leaf were used for three-dimensional (3D) analysis. Convolutional Neural Networks (CNNs) were used for identification and compared with conventional machine learning methods. For the extracted spectra, 1D CNN had a fine classification performance, and the classification accuracy could reach 98%. For RGB images, 2D CNN had a better classification performance. For HSIs, 3D CNN performed moderately and performed better than 2D CNN. On the whole, CNN performed relatively better than conventional machine learning methods. In the process of 1D, 2D, and 3D CNN visualization, the important wavelength ranges were analyzed in 1D and 3D CNN visualization, and the importance of wavelength ranges and spatial regions were analyzed in 2D and 3D CNN visualization. The overall results in this study illustrated the feasibility of using hyperspectral imaging combined with multi-dimensional CNN to detect aphid infection in cotton leaves, providing a new alternative for pest infection detection in plants.

## Introduction

Cotton is rich in cellulose and is the largest source of natural textiles (Ma et al., 2016). It has important applications in the medical field and an important position in the global economy (Rather et al., 2017). However, cotton plants are vulnerable to pests during the 6-month growth period (Wilson et al., 2018). Aphids (*Aphis gossypii* Glovers) are one of the most invasive pests in cotton plants (Wang et al., 2018). Aphids can reproduce rapidly within a few days, hiding in the lower surface of leaves and the core of young leaves. The small size and fast reproduction of aphids are the main obstacles in the control process of cotton pests. Besides, the back color of the juvenile aphids is similar to the plant color, which is not easy to be distinguished. The mature aphids have migratory and strong mobility. Once small-scale aphid pests occur in the cultivation area, the scale of the pests is likely to spread rapidly in a short time, and the cotton yield and quality will be reduced accordingly (Chen et al., 2018a).

Aphids are not only common in cotton crops, but also in traditional crops (Chen et al., 2019; Szczepaniec, 2018; Thorpe et al., 2016). Armstrong et al. (2017) studied sugarcane aphids and found suitable resistance genes in sugarcane. Hough et al. (2017) quantified the effect of temperature on the growth of soybean aphid populations. Kafeshani et al. (2018) used Taylor’s power law and Iwao’s patchiness to evaluate the spatial distribution of aphids on two citrus species (Satsuma mandarin and Thomson navel). Related scholars studied aphids from the perspectives of physiology and biochemistry, aiming to reduce the impact of aphids on crops. Combined with the experience of the Australian cotton industry, the excessive use of pesticides in the early stages would lead to high resistance in the offspring of aphids (Herron and Wilson, 2017). Therefore, quickly identifying and obtaining information on aphids, formulating efficient management strategies, and reducing the frequency of pesticide use are vital steps to increase crop yields and reduce aphids’ resistance to pesticides.

At present, the classification of pests and diseases based on imaging technology has been widely used. This technology is mainly based on pest morphology, plant texture, and morphological changes. Wang et al. (2017b) used the convolution neural network (CNN) to classify RGB images of crop pests containing 82 common pest types in a complex farmland background. Sunoj et al. (2017) used three cameras (digital single-lens reflex camera, consumer-grade digital camera, and smartphone) to take the RGB images of the front side of the leaves under infection by soybean aphids, and classified aphids according to the shape parameters. Deng et al. (2018) extracted features from RGB images of pests in complex environments, and input the features into Support Vector Machine (SVM) for recognition.

Spectroscopic technologies have been used as effective alternatives for pest and pest infection detection. Some scholars have used spectroscopy to study the degree of damage of insect pests to plants, and identify the pest infection. Moscetti et al. (2015) used near-infrared (NIR) spectroscopy to detect and remove olive fruits damaged by fruit flies. Canário et al. (2017) used spectral technology to identify tomato plants in the early stage of infection by whitefly. Basati et al. (2018) used visible/near-infrared (Vis/NIR) spectroscopy to detect wheat samples infected by pests.

Hyperspectral imaging technology combines imaging and spectroscopy techniques to detect the two-dimensional (2D) geometric space and one-dimensional (1D) spectral information of the target, and can quickly and non-destructively analyze the research object (Gao et al., 2019). Hyperspectral imaging has been widely used in plant science. It can be used to evaluate important parameters of plant health, such as nutrients, plant biomass, biological stress, and abiotic stress (Thomas et al., 2018). Plant diseases and pest detection is a significant research field of hyperspectral imaging. Previous studies have proved that hyperspectral imaging can identify the outbreak and dynamics of plant diseases and pests (Moghadam et al., 2017; Huang et al., 2018). In the application of hyperspectral imaging in pest detection as a branch of plant science research direction, the current main research includes pest identification (Liu et al., 2016) and pest infection degree classification (Lu and Ariana, 2013). Identification and segmentation of insect infected areas are one of the vital steps in pest detection. Related scholars have already identified and segmented infected areas (Tian et al., 2015). Hyperspectral images (HSIs) can provide a huge number of features, including spectral features and spatial features. As high-dimensional data, HSIs have a large amount of data information. How to mine valuable information has become a difficult problem.

Deep learning (DL) is currently a more concerning data processing method, and it has a wide range of applications in hyperspectral image processing (Signoroni et al., 2019). DL methods combined with spectral features of HSIs have been widely used in plant science, such as plant disease classification (Han and Gao, 2019). In addition, it is a new trend to select key wavelength images from HSIs and extract spatial features for disease segmentation (Feng et al., 2020). Although the performance of DL is better than conventional machine learning methods, DL methods based on spectral features or spatial features use less valuable information, ignoring the spatial features, or spectral features of HSIs. There is still room for improvement in the performance of DL. Previous studies have proved that the performance of DL methods based on spectral-spatial features is fine. A conventional method is to fuse separately extracted spectral features and spatial features (Zhao and Du, 2016; Wang et al., 2017a). Another method is to use three-dimensional (3D) CNN, whose 3D convolution kernel directly combines local spectral-spatial features (Wang et al., 2019a). At present, there are various DL architectures that combine the spectral-spatial features of HSIs, such as Resnet and DenseNet (Paoletti et al., 2019; Zhong et al., 2018).

Although DL can handle high-dimensional data, redundant features in HSIs are a huge challenge. HSIs contain much information irrelevant to the research target, which increases the computational burden, reduces the analysis efficiency, and interferes with the analysis results. Thus, dimensionality reduction (band selection and feature extraction) is a critical measure for the application of HSIs in various fields. Dimensionality reduction can be divided into linear and non-linear methods. Conventional linear methods include principal component analysis and factor analysis, which can extract features and select bands through correlation coefficients. The main non-linear dimensionality reduction methods are Isomap and Auto-Encoder, which can extract features (Kozal et al., 2013). DL, as a non-linear dimensionality reduction method, its convolutional layer can transform HSIs into low-dimensional features (Zhang et al., 2020a), and the constructed DL architectures (e.g., attention-based CNN) can select the optimal band subset (Cai et al., 2020; Lorenzo et al., 2020). Although DL can achieve better results in many cases, it is meaningful to identify the part of the input data that has a greater contribution to the research target due to a large amount of input data, so as to reduce the input of useless information (or information with low contribution) in the future research. DL visualization, which can interpret research results, is an effective way to find important features of research goals (Yosinski et al., 2015; Zhou et al., 2016). The saliency map is a DL visualization method, and its principle is to reflect the main contribution area of the input data through the gradient of the backpropagation (Simonyan et al., 2013). 2D CNN based on DL and DL visualization methods have made rapid progress in plant phenotypic stress, involving plant diseases and pests (Singh et al., 2018). Hyperspectral imaging, which can perform 1D analysis, 2D analysis, and 3D analysis, can provide spectral (1D) information, spatial (2D) information, and spectral-spatial (3D) information. At present, 1D CNN combined with hyperspectral imaging can be used for plant disease detection (Han and Gao, 2019). 3D CNN has been partially researched in plant disease detection (Nagasubramanian et al., 2019). However, there are few studies using 1D, 2D, and 3D CNN for the same plant using hyperspectral imaging, especially pest detection. Meanwhile, the use of DL visualization to find high contribution regions of input data is usually ignored.

This study aimed to explore the application of multi-dimensional CNN in aphid infection identification. In this study, hyperspectral imaging and DL were used to diagnose leaves in cotton plants infected by aphids (*A. gossypii* Glovers). Using HSIs containing a single leaf, the spectra, and RGB images were extracted. CNN models using 1D analysis in extracted spectra, 2D analysis in extracted RGB images, and 3D analysis in hyperspectral images were established, and compared with conventional machine learning methods (Logistic Regression, LR; Support Vector Machine, SVM; Nearest Neighbors, NN; Decision Tree, DT). At the same time, the results of 1D, 2D, and 3D CNN were visualized. Important wavelength ranges were discovered through the 1D and 3D CNN visualization, and important infection regions were discovered through the 2D and 3D CNN visualization.

## Materials and Methods

### Sample Preparation

Cotton plants were cultivated in greenhouses, which in the North Second District of Shihezi University, Shihezi City, Xinjiang Uygur Autonomous Region, China (86°3′34″ E, 44°18′58″ N). The cotton plants (Lumianyan 24) were planted on October 15, 2019. During the cultivation process until the seedling stage of the cotton plant, cotton plants were cultivated under two different light intensities and the same medium. It resulted in 20 high and 20 short cotton plants. Half of the cotton plants of each morphology type were inoculated with aphids, and the other half of the cotton plants without aphids were used as the control group. In the plant cultivation process, the leaves in cotton plants would not curl up and deform under the infection of aphids, which was suitable for shooting. At the same time, the blade size of leaves was similar, which was conducive to modeling. During the plant cultivation, the highest temperature of the culture environment was 28.5°C, the lowest temperature was 15.5°C, and the average temperature was 20.85°C. The highest relative humidity was 56%, the lowest relative humidity was 32%, and the average relative humidity was 44.475%.

**FIGURE 1:**
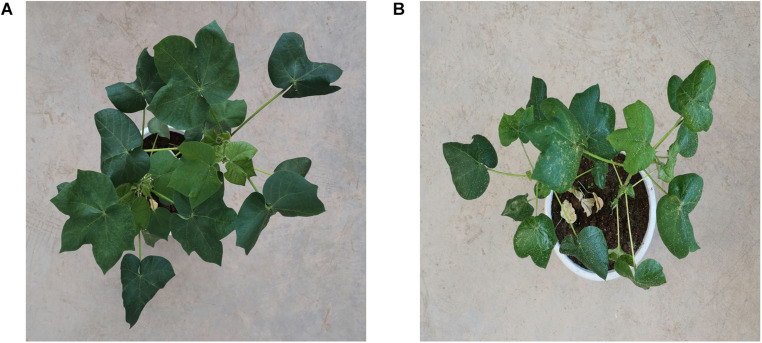
**(A)** An image taken from the overhead view of healthy cotton plants. **(B)** An image taken from the overhead view of cotton plants infected by *Aphis gossypii* Glovers.

As shown in Figure 1, RGB images taken from the overhead view of infected cotton plants and healthy cotton plants are shown. Some areas of cotton leaves infected by aphids are white. The reason is that the area is covered with aphid secretions, which is a carbohydrate that reflects most of the light.

### Hyperspectral Image Acquisition and Preprocessing

The research object of this study was healthy leaves and infected leaves and the HSIs were taken from December 5, 2019 to December 25, 2019, with an interval of 5 days. For each sampling and shooting time, cotton plants were destructively sampled, and the front of the leaves was shooted. To reduce the interference of biochemical factors such as the open state of leaves, the shooting time was fixed at around 14:00 (UTC/GMT + 08:00).

In this study, the Vis/NIR hyperspectral imaging systems were composed of four modules, including an imaging module, an illumination module, a lifting module, and a software module. The imaging module was SOC 710VP camera (Surface Optics Corporation, San Diego, CA, United States). The camera had a push broom and dual CCD detectors. When the sample was taken at a fixed position, the SOC 710VP hyperspectral camera used the internal translation push-broom mechanism to scan samples. The HSI size was 128 wavebands × 520 pixels × 696 pixels, each pixel contained the full spectrum in the range of 376–1044 nm with the spectral resolution of 5 nm. The lighting module was composed of two halogen lamps with a power source of 75 W. The lifting platform module placed the shooting object, and the imaging module could fully capture the shooting object by lifting. The software module was used to control HSI acquisition. The shooting integration time of the hyperspectral camera was 25 ms, the aperture value was F1.4, and the shooting height was 86 cm. During the HSI acquisition process, the imaging conditions and system parameters were kept unchanged. After HSI acquisition, the original HSIs were calibrated to reflectance images according to Equation 1.

(1) $$I_{c}=\frac{I_{r}}{2I_{g}}$$

Where *I*_*c*_ is the reflectance image, *I*_*r*_ is the original image, and *I*_*g*_ is the gray (combined with 50% black and 50% white) reference image.

**FIGURE 2:**
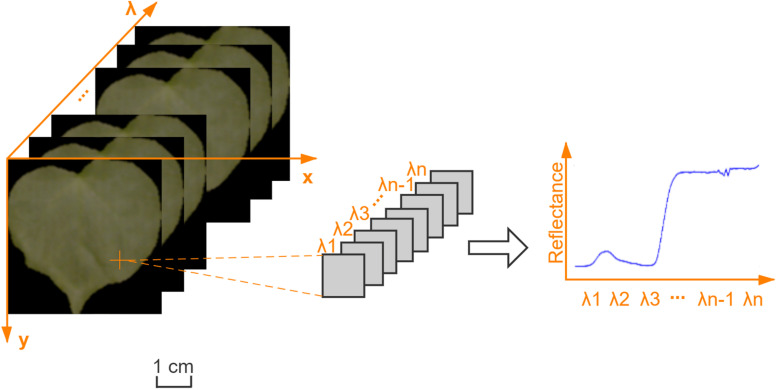
The three-dimensional data cube of a cotton leaf hyperspectral image.

The Savitzky–Golay smoothing filter (the kernel size was 5 × 5 × 5, the polynomial order was 3, the filter calculated the filtered value at the central node of the kernel) was used to reduce the random noise on the reflectance HSIs. The 3D data cube of a cotton leaf HSI is shown in Figure 2.

### Data Set Construction

In this study, a total of 256 HSIs of infected leaves and healthy leaves were collected. Considering that wavelengths under 450 nm and over 1000 nm have more noise, as a limitation of the sensor (Malenovsky et al., 2006). Only the wavelengths in the range of 461–988 nm were studied.

The software provided by SOC was used to synthesize RGB images at wavelengths of 461, 548, and 698 nm in HSIs.

In HSIs, the area containing a single leaf was regarded as a region of interest (ROI). The pixel-wise spectra (461–988 nm) in the ROI were extracted and averaged to represent the sample. Due to the average spectra contains redundant and collinear information, and the use of the first derivative can suppress background information (Jin and Wang, 2016). The first derivative spectra of the average spectra were used.

### Data Analysis Methods

#### Conventional Machine Learning Methods

Logistic regression (LR) is a generalized linear regression analysis model (Stoltzfus, 2011). LR uses the Logistic Sigmoid function to convert the output into a probability value to predict the label. The basic LR model deals with binary classification problems. For the LR model, the regularization parameter C is used to solve the model fitting problem. In this study, the optimization range of C was in [10^−5^–10^5^].

Support Vector Machine (SVM) is a common classification algorithm used for supervised learning (Jian et al., 2016). The principle of SVM is to find the hyperplane with the largest interval in the feature space. SVMs are currently divided into linear SVM, polynomial SVM, radial basis function (RBF) SVM, and sigmoid SVM. The SVM using the “linear” kernel function is essentially a linear classifier, similar to LR. To compare with LR, the kernel optimization range was in (“polynomial,” “sigmoid,” “RBF”) in SVM. For the SVM model, the regularization parameter C and the kernel coefficient γ are used to solve the model fitting problem. In this study, the optimization range of C and γ were all in [10^–5^–10^5^].

Nearest Neighbors (NN) is a widely used pattern recognition method (Zhang et al., 2018). The principle of NN is to find training samples that meet the first K shortest distances of distance test samples and predict the label based on these training samples. In this study, the optimization range of K was in [1, 30].

Decision Tree (DT) is a supervised learning method for classification (Wang et al., 2019b). Its purpose is to create a model that can learn simple decision rules from data features. DT uses the rules to predict the label of a test sample. DT learns data through if-then-else decision rules and estimates the label of the predicted sample. The deeper the decision tree, the more complex the decision rules and the better the fit to the training samples. For the DT model, the parameter max_depth is used to limit the depth of the tree. In this study, the optimization range of max_depth was in [1, 30].

#### Convolutional Neural Network

Convolutional Neural Network (CNN) is a neural network based on convolutional layers. The CNN model usually consists of five parts as input, convolution, pooling, dense connection, and output. There are differences in these five parts of the current mainstream CNN models (Khan et al., 2019). Since Resnet solves the problem of network degradation in DL, it becomes the backbone network for subsequent research (Sanchez-Matilla et al., 2020; Veit and Belongie, 2020; Zhang et al., 2020b). In this study, Resnet-18 was used as the backbone network to construct CNNs (He et al., 2016).

For spectra, the network layers of Resnet-18 were adjusted. The process of sliding windows of each network layer for 2D analysis was adjusted to the process of sliding windows for 1D analysis. For RGB images, Resnet-18 was directly used. For HSIs, the classification model suitable for 3D analysis was designed. The residual blocks were used in the model. The residual block allowed the training of the deep network to proceed smoothly. The main reason was that the stack of the residual block could effectively return the gradient, and the skip connection was added based on the stack. Since HSIs had three dimensions (depth, height, and width), and the amount of data was large, the network structure based on 3D CNN should not be complicated. Otherwise, there would be insufficient computing power.

**FIGURE 3:**
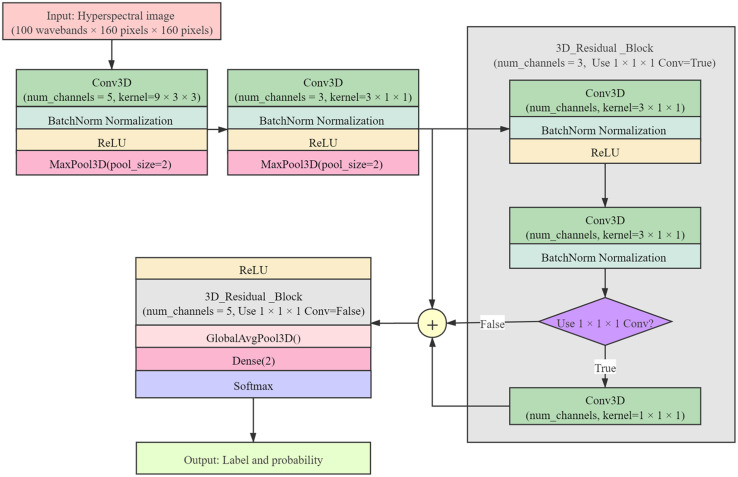
3D CNN architecture.

In this study, 3D CNN consisted of two convolutional layers, two batch normalization layers, two max-pooling layers, two residual blocks, a global average pooling layer, and a dense layer, followed by a Softmax layer. Since the size of a larger convolution kernel will improve the performance of the network, smaller size of the convolution kernel will increase the convergence speed of the network (Cai et al., 2018; Tan and Le, 2019). Considering the convolution kernel size as a compromise, the convolution kernel with the size of 9 × 3 × 3 was used as the first convolutional layer, the convolution kernel with the size of 3 × 1 × 1 was used as the second convolutional layer, and the number of channels was 5 and 3 in turn. The Rectified Linear Unit (ReLU) was used as the activation function of the convolution output. The size of max-pooling layers was 3. The 1 × 1 × 1 convolutional layer was used in the first residual block and not used in the second residual block. The number of channels in two residual blocks was 3 and 5 in turn. 3D CNN architecture is shown in Figure 3.

The CNN models could be used for data dimensionality reduction (Song et al., 2019). The CNN models non-linearly map high-dimensional data to low-dimensional space. In this study, the RGB images and hyperspectral images reduced by the CNN models were used for modeling based on conventional machine learning methods. The global pooling layer features in the CNN models were used in the modeling process of conventional machine learning methods.

#### Saliency Map of Convolutional Neural Network

The saliency map is a CNN visualization method that can reflect the impact of each data element on the classification results. In this study, the saliency map visualization method proposed by Simonyan et al. (2013) was used. When the sample label was correctly predicted, each element in the data would have a corresponding contribution value, and the magnitude of the contribution value reflected the importance of the elements. The contribution value was visualized by saliency maps, which could effectively observe the important regions of the sample identified by CNN.

Given a data *D*_0_ of category *c* in the test set, after being classified by the CNN model, the score value *S*_*c*_ will be obtained. If the predicted category is consistent with the true category, the weight can be calculated. The approximate calculation process is carried out according to Equation 2.

(2) $$w=\text{abs}\left(\frac{\partial S_{c}}{\partial D}|D_{0}\right)$$

Where *w* is the absolute value of the derivative of score *S*_*c*_ concerning data *D*_0_, and *w* is valid only when the predicted category is consistent with the true category.

In the case of a HSI, the wavelength variable *v* of the pixel (*i*, *j*) of the image I corresponds to the *w* element whose index is *h*(*i*, *j*, *v*). To obtain the contribution value of a single category for each pixel (*i*, *j*), the maximum value *M* of *w* on all wavelength variables is used, as shown in Equation 3.

(3) $$M={\text{max}}_{v}\left(w_{h_{\left(i,j,v\right)}}\right)$$

In this study, another interpretation method was visualized for the saliency map. As shown in Equation 4, the cumulative contribution C of wavelengths in the test set is calculated, and the L1-norm is used to normalize by column.

(4) $$C=\frac{\sum_{j}\in \left(1,2,\ldots S\right)M_{j}}{||\sum_{j}\in \left(1,2,\ldots S\right)M_{j}|{|}_{1}}$$

Among them, *M*_*j*_ is the saliency map of the *j*th sample, and the number of samples is *S*.

To compare with the spectral wavelengths and observe the wavelengths in the hyperspectral images with the high contribution rate, the L1-norm visualization method proposed by Nagasubramanian et al. (2019) was used in this study.

#### Model Evaluation

To better evaluate the performance and stability of the models, the training set, validation set, and test set of HSIs in each shooting period were divided according to the ratio of 3:1:1. The division of spectral data set, RGB image data set, and HSI data set is shown in Table 1. The first derivative spectra, RGB images, and HSIs of all samples had a one-to-one correspondence in each set. In each set, the number of samples of each class was almost equal, with slight differences due to the number of leaves in different cotton plants. Due to the small amount of data in different periods, it was impossible to explain the best period to detect infection. However, the number of healthy samples and infected samples in general could be used to model and explore important wavelengths and regions.

**TABLE 1:** The division of data set.

Data Set Type	Training	Validation	Test
First derivative spectra	75/78^a^	26/25	24/28
RGB images	75/78	26/25	24/28
Hyperspectral images	75/78	26/25	24/28

*^a^The number of category samples is expressed as: the number of healthy samples/number of infected samples.*

Batch normalization could speed up model convergence and shorten the model building time (Ioffe and Szegedy, 2015). In this study, batch normalization was used for all sample sets before training.

Bayesian optimization algorithm (BOA) was used in the parameter optimization process of conventional machine learning methods (Pelikan and Goldberg, 1999). The classification accuracy was used to evaluate the performances of each model, which was calculated as the ratio of the number of correctly classified samples to the total number of samples. The training set was learned by the models, and the model parameters were optimized by BOA 200 times. The models with the highest prediction accuracy in the validation set were saved and evaluated in the test set.

In this study, for CNN models, the batch size was 10 and the epoch was 200 while training the models, the optimization algorithm was set to SGD, the initial learning rate was set to 0.1, and the learning rate was gradually adjusted to 0.01 during the training process. The CNN models were trained from scratch and initialized using the Xavier method. During the training process, the CNN models with the best fit in the validation set, and the lowest loss value were selected, and the test set was used to evaluate the models.

#### Software and Hardware

In this study, Python scripting language (version 3.7.6, 64 bit) was used for numerical calculations. Conventional machine learning methods were implemented on the python library package scikit-learn (version 0.23.1). The CNN models were built on the MXNet (version 1.5.0) framework (Amazon, Seattle, WA, United States). All data analysis procedures were implemented on a computer with 16 GB of RAM, the NVIDIA GEFORCE GTX 1080Ti GPU, and the Intel Core i7-9700K CPU.

## Results

### Spectral Profiles

**FIGURE 4:**
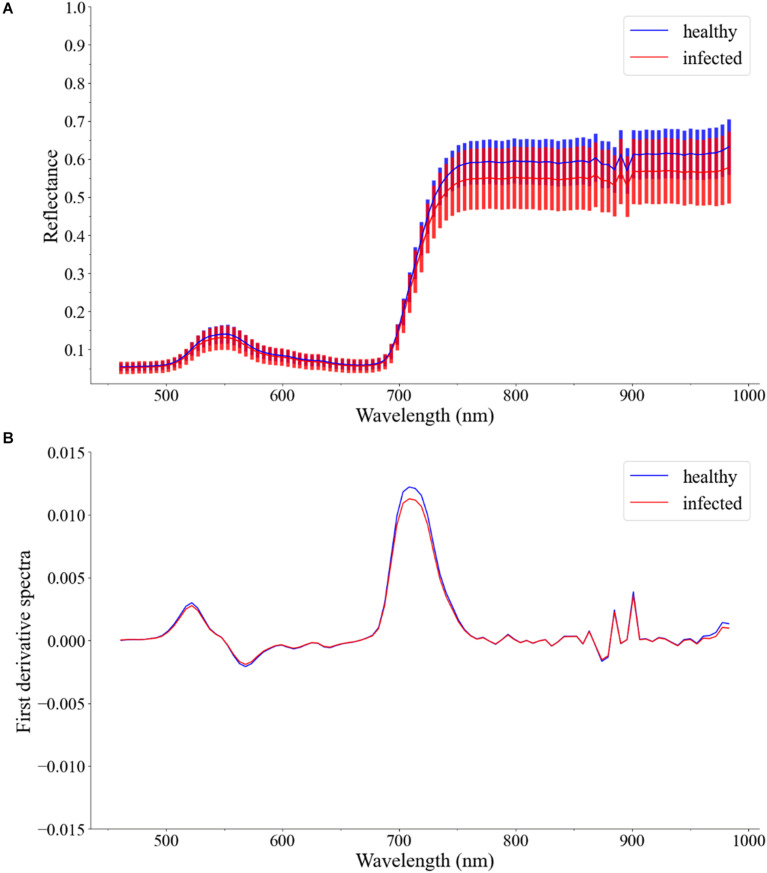
**(A)** Vis/NIR average spectra (461–988 nm) and standard deviation for healthy leaves and infected leaves. **(B)** First derivative spectra of average spectra.

In this study, the first derivative spectra were used to build the models. Figure 4A shows the Vis/NIR average spectra (461–988 nm) and standard deviation for each class of leaves. Figure 4B shows the first derivative spectra of average spectra.

For the Vis/NIR average spectra, the reflectance of healthy leaves was slightly higher. There was a large overlap in the standard deviation areas of the reflectance of healthy leaves and infected leaves. For the first derivative spectra of average spectra, there was no difference between healthy leaves and infected leaves, except around 720 nm. In general, the spectra of different types of samples cannot provide clear enough discrimination. Therefore, other classification methods should be considered.

### Classification Models

For conventional machine learning models, the features of the global pooling layer reduced by CNN models were used for modeling and evaluation. The CNN models used for dimensionality reduction were trained from scratch and could completely predict the categories of all samples. For CNN models, the images were resized to reduce the influence of sample shape on DL. The resized RGB image size was 160 pixels × 160 pixels and the resized HSI size was 100 wavebands (461–988 nm) × 160 pixels × 160 pixels. The classification results of each model are shown in Table 2. The classification results of some other conventional classification methods are reflected in Supplementary Table 1.

**TABLE 2:** Classification accuracy of the conventional machine learning methods and convolutional neural network (CNN).

Data set type	Methods	Category values	Training			Validation			Test		
			0	1	Accuracy	0	1	Accuracy	0	1	Accuracy (%)
First derivative spectra	LR	0^a^	71	4		22	4		21	3	
		1	7	71		4	21		4	24	
		Total			92.81			84.31			86.54
	SVM	0	74	1		22	4		15	9	
		1	2	76		2	23		5	23	
		Total			98.04			88.24			73.08
	NN	0	75	0		17	9		14	10	
		1	0	78		7	18		6	22	
		Total			100.00			68.63			69.23
	DT	0	67	8		14	12		20	4	
		1	0	78		8	17		5	23	
		Total			94.77			60.78			82.69
	CNN	0	75	0		26	0		24	0	
		1	0	78		0	25		1	27	
		Total			100.00			100.00			98.08
RGB images	LR	0	75	0		26	0		24	0	
		1	0	78		0	25		8	20	
		Total			100.00			100.00			84.62
	SVM	0	75	0		26	0		23	1	
		1	0	78		0	25		9	19	
		Total			100.00			100.00			80.77
	NN	0	75	0		26	0		24	0	
		1	3	75		1	24		13	15	
		Total			98.04			98.04			75.00
	DT	0	75	0		24	2		21	3	
		1	0	78		0	25		9	19	
		Total			100.00			96.08			76.92
	CNN	0	75	0		26	0		24	0	
		1	0	78		0	25		8	20	
		Total			100.00			100.00			84.62
Hyperspectral images	LR	0	75	0		23	3		21	3	
		1	0	78		4	21		6	22	
		Total			100.00			86.27			82.69
	SVM	0	75	0		26	0		21	3	
		1	0	78		2	23		5	23	
		Total			100.00			96.08			84.62
	NN	0	75	0		26	0		24	0	
		1	15	63		8	17		11	17	
		Total			90.20			84.31			78.85
	DT	0	75	0		22	4		18	6	
		1	0	78		4	21		4	24	
		Total			100.00			84.31			80.77
	CNN	0	70	5		25	1		22	2	
		1	6	72		3	22		4	24	
		Total			92.81			92.16			88.46

*^a^0 means the label of the healthy leaves, 1 means the label of the infected leaves.*

For the first derivative spectra, the CNN model performed best, with an accuracy rate of 98.08% in the test set. For RGB images, LR and CNN model performed best, with an accuracy rate of 84.62% in the test set. For HSIs, the CNN model performs best, with an accuracy rate of 88.46% in the test set.

For the first derivative spectra, RGB images, and HSIs, CNN performed best, and LR, SVM, and DT model perform worse in turn, and the worst was the NN model.

For the performance of the same model in different datasets, most models performed best in the first derivative spectra, followed by HSIs, and the worst performance in RGB images.

### Visualization of Convolutional Neural Network

**FIGURE 5:**
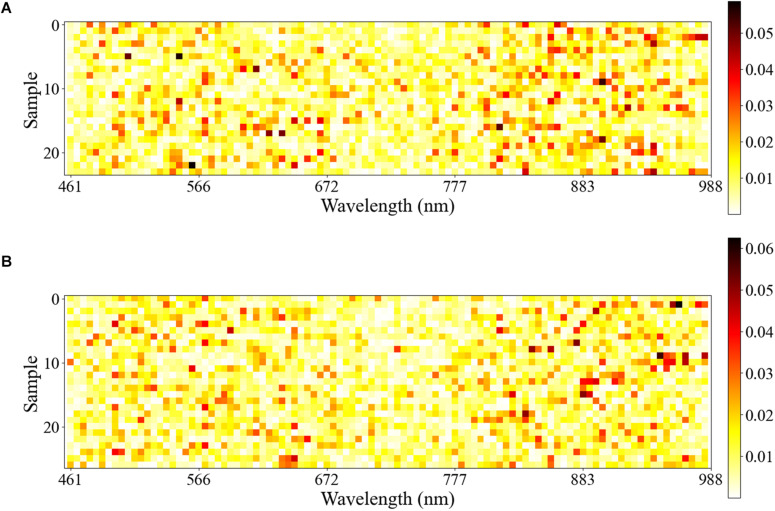
The row coordinate represents the wavelength, and the ordinate represents the sample correctly predicted by 1D CNN. The depth of each row of color represents the importance of the wavelength of the corresponding sample in the process of identifying *A. gossypii* Glovers infection. **(A)** Visualization of the first derivative spectra test set of healthy leaves. **(B)** Visualization of the first derivative spectra test set of infected leaves.

In this study, the test set used for 1D CNN was visualized by Equations 2 and 3. The samples of healthy leaves and infected leaves were normalized so that the sum of the wavelength contribution value of each sample was 1. The normalized results were displayed visually. As shown in Figure 5, the row coordinates represent the wavelength, and the ordinate represents the sample.

For all the samples in the test set, the wavelength with the largest contribution was concentrated in the NIR wavelength range of 750–950 nm, followed by the Vis wavelength range of 460–660 nm.

**FIGURE 6:**
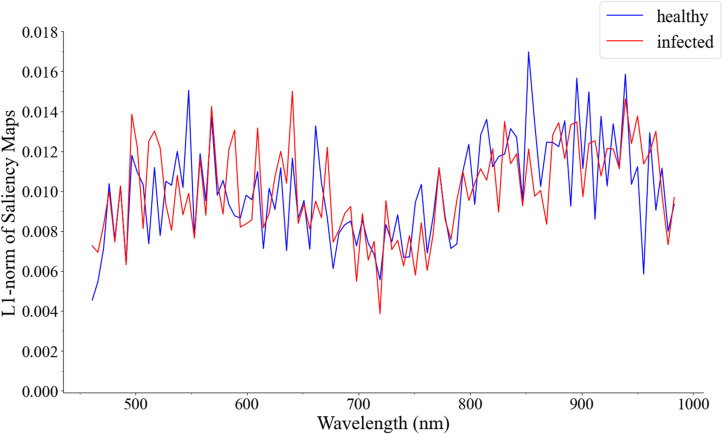
Visualization of the cumulative contribution of wavelengths of samples correctly predicted in the 1D CNN in the first derivative spectra test set.

The saliency maps of healthy leaves and infected leaves were visually explained in Equation 4. The visualization results are shown in Figure 6.

In Figure 6, the cumulative contribution value is very low in the red-side spectral range of 660–750 nm. The most contributory wavelength ranges were concentrated in 750–950 nm, followed by 460–660 nm, which also explains the important wavelength ranges of the saliency maps. The 460–600 nm wavelengths and 750–950 nm wavelengths of the spectral data set were respectively intercepted, and 1D CNN was used for re-modeling. As a result, in the performance of the test set, the accuracy of 460–600 nm wavelengths was 71.15%, and the accuracy of 750–950 nm wavelengths was 90.38%. The results confirmed the effectiveness of the visualization method.

**FIGURE 7:**
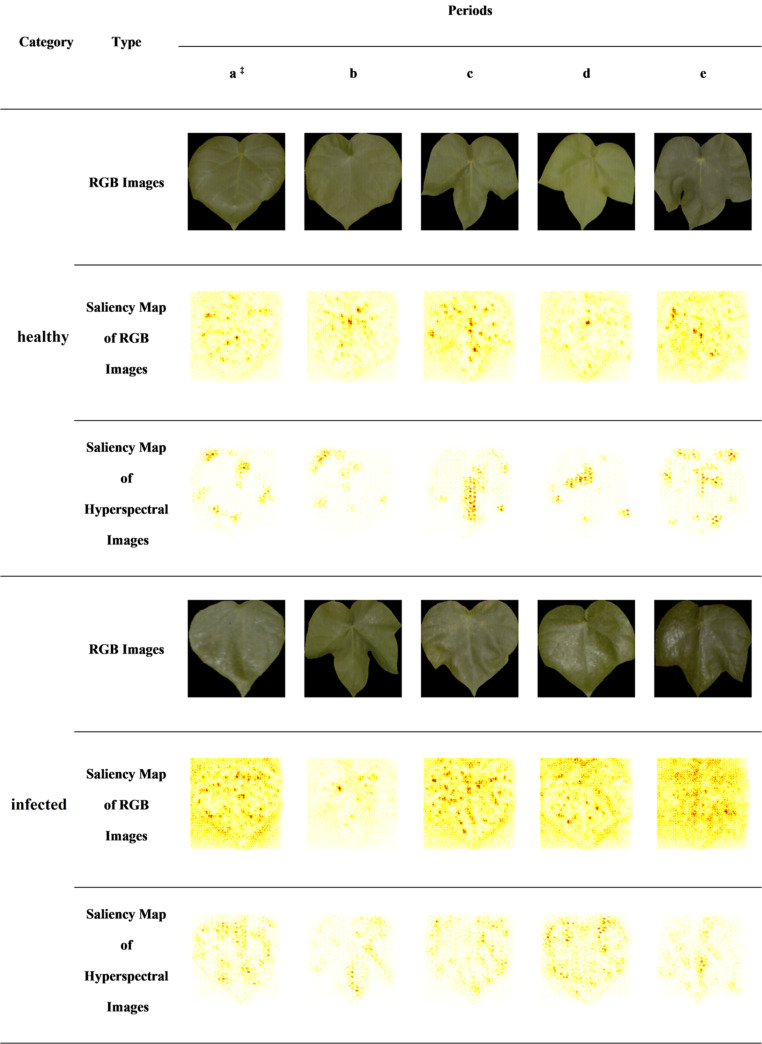
CNN-based saliency map of RGB images and hyperspectral images. The saliency maps of RGB images are based on 2D CNN, and the saliency maps of hyperspectral images are based on 3D CNN. In each saliency map, the features of the darker regions have a greater impact on the identification results. ^‡^a–e indicates the five periods of dataset collection in turn.

In this study, the RGB images and the HSIs were selected from the test set of healthy leaves and infected leaves (one-to-one correspondence between RGB image and HSI samples). As shown in Figure 7, the saliency maps are visualized on the test set, which includes the samples of two classes in five periods, and the labels of all samples are correctly predicted.

Cotton leaves infected by aphids will attach aphid secretions. Aphid secretion is a carbohydrate product secreted by *A. gossypii* Glovers, which has a high reflection effect. The secretion of aphids causes the leaves to turn white. At the same time, the texture of real leaves under the infection of aphids is changed. It could be seen from the RGB images that part of the infected leaves was white.

In the saliency map of the 2D CNN, it was found that the pixel area with the largest contribution value was concentrated on the aphids’ secretions (leaf whiteness) and leaf textures in infected leaves. 2D CNN looked for the presence of these areas that affect classification in healthy leaves. And there was no whitening in some areas of healthy leaves caused by aphid secretions. Therefore, in the saliency map of 2D CNN, there were obviously few areas with larger contribution values, and the leaf textures were mainly displayed in healthy leaves.

In 3D CNN visualization, the pixel area with the largest contribution values was always concentrated near the leaf veins and leaf edges.

**FIGURE 8:**
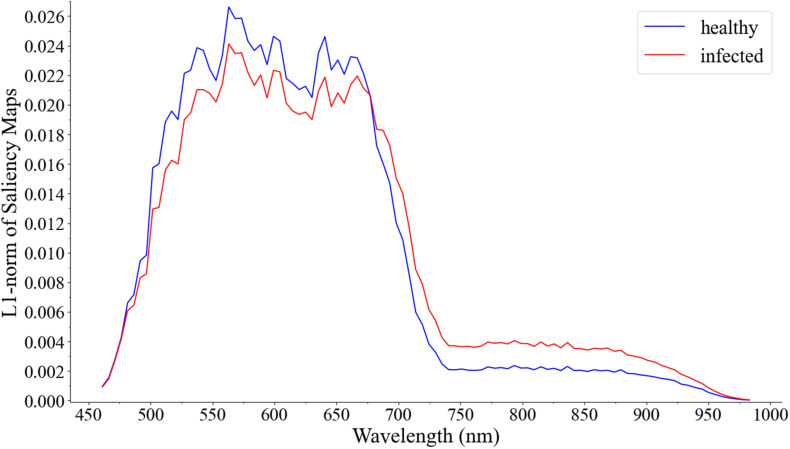
Visualization of the wavelength contribution of the hyperspectral image test set.

By using the method proposed by Nagasubramanian et al. (2019), the visualization result of the wavelength contribution rate of HSIs is shown in Figure 8.

The results showed that the 3D CNN model was not sensitive to the 750–950 nm wavelengths in HSIs, and had excellent sensitivity to the 460–600 nm wavelengths. In HSIs, the pixel area with the largest contribution rate depends on the 460–600 nm wavelengths. The 460–600 nm wavelengths and 750–950 nm wavelengths of the HSI set were respectively intercepted, and 3D CNN was used for re-modeling. As a result, in the performance of the test set, the accuracy of 460–600 nm wavelengths was 88.46%. And the accuracy of 750–950 nm wavelengths was 53.85%, which was similar to blind guessing. The results confirmed the effectiveness of the visualization method. It could also indicate that the 3D CNN model paid attention to the area around the leaf veins and the leaf edges in the 460–600 nm wavelengths of the HSIs.

## Discussion

Aphids not only appear on cotton crops but also often appear on other traditional crops. Aphids affect the growth and development of crops, which in turn affect the quality and yield of agricultural products grown on crops. From a physiological point of view, the selection of aphid-resistant crops is one of the effective measures to reduce the impact of aphids, but the process of crop selection and breeding takes a long time (Cabral et al., 2018; Zhan et al., 2020). From a chemical and physical point of view, the infection caused by aphids can be captured by the imaging system (Chen et al., 2018b). Currently, there are few studies on aphid detection based on near-ground object hyperspectral imaging. In this study, the Vis/NIR hyperspectral imaging system was used to detect aphid infection and direct sensory monitoring of aphid infection. It can provide a reference for the direct control of aphids on crops.

Due to hyperspectral imaging can obtain spatial and spectral information of the research object, both spatial and spectral information in hyperspectral imaging can be used to detect pest infection and disease infection (Ahmad et al., 2018). Therefore, the spectral information and spatial information based on hyperspectral imaging have been introduced in previous research. Previous studies have determined that using spectral information in hyperspectral imaging is highly effective for detecting pest infection and disease infection (Rady et al., 2017). It is feasible to use the average spectra of hyperspectral imaging or the pixel-wise spectra for 1D analysis and detection of infection. Zhou et al. (2019) used average spectra to detect infection. Qiu et al. (2019) used the spatial information of spectral imaging and used the pixel-wise spectra to detect the infected area. However, the 1D analysis does not make full use of the spatial information of hyperspectral imaging. Due to a large amount of hyperspectral imaging data, the selection of key wavelength images is important for the use of spatial information (Li et al., 2019). According to the 2D analysis of the key wavelength information, the plant infection area can be marked with higher precision. However, 1D analysis and 2D analysis are not sufficient for the mining and utilization of spatial information or spectral information. The 3D analysis makes effective use of spatial information and spectral information, and it has a few applications in plant disease monitoring. At present, there are few studies on simultaneous 1D analysis, 2D analysis, and 3D analysis for the same infection. In this study, 1D analysis, 2D analysis, and 3D analysis were used to detect aphid infection in cotton leaves, and the multi-dimensional detection results were all good. On the whole, 1D analysis is worthy of consideration for rapid detection of infection, and 2D analysis and 3D analysis can be used to detect the infected area.

Currently, conventional machine learning methods are used to monitor plant diseases and insect pests. Meanwhile, the DL method has been widely used in the monitoring of plant diseases and insect pests. 1D DL and 2D DL are widely used in plant diseases and insect pest detection. 3D DL has been partially applied in the monitoring of plant diseases and insect pests. However, the DL method has not been reasonably explained in the detection of plant diseases and insect pests. In this study, the saliency map was used to visualize the DL model. Through visualization, important wavelengths and spatial regions were discovered. The important wavelengths and spatial regions were consistent with actual conditions. Overall, the visualization of DL provides new ideas for the interpretation of the application of plant pests and disease detection in DL in the future.

Overall, the classification results of the 1D analysis, 2D analysis, and 3D analysis were fine. In the field of spectroscopy, DT and NN models were prone to overfitting problems, which may be the reason for the poor results (Liu et al., 2008; Mandrell et al., 2020). RGB images lost a lot of spectral information and only contain color information, which may be the reason for their poor performance. Due to the computing power of the computer in this study, HSIs contain a lot of spectral information and spatial information, but the 3D CNN model may not make full use of information. For the first derivative spectra, while the spatial information was lost, the spectra were simple but contain enough information, and most models were easy to learn data features under the existing computer computing power, which may be the reason for the outstanding effect. Considering the impact of the training set size on the overall performance of the investigated classification methods, the size of the validation and test sets were kept constant, and the training set size was sequentially expanded from 25/27, which was the same size as the validation and test sets, to 75/78 (the number of healthy samples/number of infected samples). The results are shown in Supplementary Table 2. The overall performance was poor when the training set size was 25/27, and when the training set size was 50/52, the overall performance approximated the overall performance of the training set size of 75/78. With the increase of training set size, the overall performance grew slowly. The overall performance was likely to be the best when the training set size was 75/78.

Aphids pierce and suck plant tissues on tender leaves, tender stems, buds, and floral organs, and other young parts of the plant, and suck the juice, which will make the veins and leaves green, yellow, white, or thin (Dubey et al., 2013). These will cause changes in the color, texture, and spectral reflectance of the leaves. In the process of visualization, the saliency maps showed that the 1D CNN model was interested in the 750–950 nm wavelengths, followed by the spectral range of 460–660 nm. The 2D CNN could capture the color and texture characteristics of the leaves, and the model itself did not notice the aphids. The main interest spectral range of the 3D CNN model was 460–660 nm, and its interest area was around the leaf veins and the leaf edges. Combined with the L1-norm visualization of HSIs, it was found that 3D CNN was not interested in the spectral range of 750–950 nm. Besides, for the 1D and 3D CNN models, the datasets in the range of 460–660 nm and 750–950 nm were re-modeling, respectively, and the test results were consistent with the visualization results, indicating the effectiveness of the visualization methods. It can be used for wavelength selection. However, 1D CNN and 3D CNN models had differences in the regions of interest of the corresponding data sets. The reason may be that the 1D CNN model captured the information of the overall structural change of the blade and the information of the spectral reflectance change in the NIR spectral range. For the 3D CNN model, it could capture the changes in the spectral reflectance of the leaves in the Vis spectral range of the HSIs, but it was difficult to capture the NIR information about the changes in the internal chemical composition of the leaves.

In this study, only the infected leaves and the healthy leaves in cotton plants were studied. Since HSIs were obtained in a greenhouse, the interference factors affecting the HSIs were controllable. In the controllable environment, typical infected samples and healthy samples were obtained, and the difference between the two categories of HSIs was large. However, there are many uncontrollable factors in field conditions, which cause uncertainty and variations in samples. The differences between healthy and infected samples might not be so large, and a large number of field experiment samples should be studied in future research. Our study provided an initial assessment of pest detection in cotton, and provide a potential method for rapid and non-invasive pest detection. In future researches, the developed method will be validated and updated based on the in-field experiments.

## Conclusion

In this study, the Vis/NIR hyperspectral imaging system (376–1044 nm) and machine learning methods were used to identify aphid infection in cotton leaves. Spectra, RGB images, and hyperspectral images containing a single leaf were used to build classification models. Spectra did not contain spatial information. However, the spectral information in the spectra was simple but rich, which was conducive to the learning of existing computing power and models, and had achieved excellent results. The RGB images and the hyperspectral images contained spatial information, and the characteristics of the spatial region that affect the classification results could be found. Compared with the RGB images, the hyperspectral images contained a lot of spectral information. The classification results of the 3D CNN used to identify aphid infection were better than 2D CNN and worse than 1D CNN. It was recommended that 1D CNN could be used to quickly and accurately identify aphid infection. In the visualization of 1D CNN, it was found that the important spectral regions of the spectra were concentrated in the Vis (460–660 nm) and NIR (750–950 nm) range. In the visualization of 2D CNN and 3D CNN, the spatial regions of cotton leaves changed after aphid infection were found. 2D CNN could be used to find aphid infection areas. 3D CNN combined features of 1D CNN and 2D CNN, it could be used to discover the aphid infection area while discovering important spectral regions. Based on the exploration of CNNs in multiple dimensions on aphid infection, the effects of CNNs in various dimensions were compared, which provided data reference for related scholars and provided new ideas for future research on pest infection.

## Data Availability Statement

The original contributions presented in the study are publicly available. This data can be found here: https://doi.org/10.6084/m9.figshare.13668314.

## Author Contributions

TY and JL prepared materials and used the hyperspectral imaging system to obtain hyperspectral images. TY and WX wrote the manuscript. TY completed the development and implementation of methods. LD tested the results. WX, PG, and CZ designed the experiment. CZ made guidance for the writing of the manuscript. PG and CZ provided funding for this work. XL reviewed the initial design of the experiments and made comments. All authors contributed to the article and approved the submitted version.

## Conflict of Interest

The authors declare that the research was conducted in the absence of any commercial or financial relationships that could be construed as a potential conflict of interest.
