# Supplementary material for: Combining Multi-Dimensional Convolutional Neural Network (CNN) With Visualization Method for Detection of Aphis gossypii Glover Infection in Cotton Leaves Using Hyperspectral Imaging
Source: Front Plant Sci. 2021 Feb 15;12:604510. doi: 10.3389/fpls.2021.604510 (PMC7917247; doi:10.3389/fpls.2021.604510)
Supplement: Supplementary file 1 [file Data_Sheet_1.docx]

Supplementary Material

**Supplementary Table 1. Classification accuracy of the conventional machine learning methods.**

| **Data Set Type** | **Methods** | **Category Values** | **Training** | | | **Validation** | | | **Test** | | |
| --- | --- | --- | --- | --- | --- | --- | --- | --- | --- | --- | --- |
|  |  |  | **0** | **1** | **Accuracy (%)** | **0** | **1** | **Accuracy (%)** | **0** | **1** | **Accuracy (%)** |
| **First Derivative Spectra** | **Partial Least Squares-Discriminatory Analysis** | **0** **^§^** | 75 | 0 |  | 22 | 4 |  | 21 | 3 |  |
|  |  | **1** | 3 | 75 |  | 4 | 21 |  | 4 | 24 |  |
|  |  | **Total** |  |  | 98.04 |  |  | 84.31 |  |  | 86.54 |
|  | **Stochastic Gradient Descent** | **0** | 74 | 1 |  | 22 | 4 |  | 15 | 9 |  |
|  |  | **1** | 2 | 76 |  | 2 | 23 |  | 5 | 23 |  |
|  |  | **Total** |  |  | 98.04 |  |  | 88.24 |  |  | 73.08 |
|  | **Gradient Boosting** | **0** | 75 | 0 |  | 22 | 4 |  | 15 | 9 |  |
|  |  | **1** | 0 | 78 |  | 4 | 21 |  | 5 | 23 |  |
|  |  | **Total** |  |  | 100.00 |  |  | 84.31 |  |  | 73.08 |
| **RGB Images** | **Partial Least Squares-discriminatory Analysis** | **0** | 75 | 0 |  | 26 | 0 |  | 24 | 0 |  |
|  |  | **1** | 0 | 78 |  | 0 | 25 |  | 8 | 20 |  |
|  |  | **Total** |  |  | 100.00 |  |  | 100.00 |  |  | 84.62 |
|  | **Stochastic Gradient Descent** | **0** | 75 | 0 |  | 26 | 0 |  | 23 | 1 |  |
|  |  | **1** | 0 | 78 |  | 0 | 25 |  | 9 | 19 |  |
|  |  | **Total** |  |  | 100.00 |  |  | 100.00 |  |  | 80.77 |
|  | **Gradient Boosting** | **0** | 75 | 0 |  | 26 | 0 |  | 24 | 0 |  |
|  |  | **1** | 3 | 75 |  | 1 | 24 |  | 13 | 15 |  |
|  |  | **Total** |  |  | 98.04 |  |  | 98.04 |  |  | 79.62 |
| **Hyperspectral images** | **Partial Least Squares-Discriminatory Analysis** | **0** | 75 | 0 |  | 23 | 3 |  | 24 | 0 |  |
|  |  | **1** | 0 | 78 |  | 2 | 23 |  | 11 | 17 |  |
|  |  | **Total** |  |  | 100.00 |  |  | 90.20 |  |  | 78.85 |
|  | **Stochastic Gradient Descent** | **0** | 75 | 0 |  | 26 | 0 |  | 19 | 5 |  |
|  |  | **1** | 0 | 78 |  | 2 | 23 |  | 11 | 17 |  |
|  |  | **Total** |  |  | 100.00 |  |  | 96.08 |  |  | 69.23 |
|  | **Gradient Boosting** | **0** | 75 | 0 |  | 26 | 0 |  | 21 | 3 |  |
|  |  | **1** | 10 | 68 |  | 8 | 17 |  | 5 | 23 |  |
|  |  | **Total** |  |  | 93.46 |  |  | 84.31 |  |  | 84.62 |

^§^ 0 means the label of the healthy leaves, 1 means the label of the infected leaves.

**Supplementary Table 2. Classification accuracy of classifiers based on different training set sizes.**

| **Data Set Type** | **Methods** | **Training Set Sizes** | **Training Accuracy** | **Validation Accuracy** | **Test Accuracy** |
| --- | --- | --- | --- | --- | --- |
| **First Derivative Spectra** | **LR** | **25/27 ^§^** | **84.97** | **74.51** | **69.23** |
|  |  | **50/52** | **88.89** | **88.24** | **82.69** |
|  |  | **75/78** | **92.81** | **84.31** | **86.54** |
|  | **SVM** | **25/27** | **88.24** | **78.43** | **67.31** |
|  |  | **50/52** | **90.20** | **86.27** | **71.15** |
|  |  | **75/78** | **98.04** | **88.24** | **73.08** |
|  | **NN** | **25/27** | **91.50** | **78.43** | **63.46** |
|  |  | **50/52** | **93.46** | **74.51** | **63.46** |
|  |  | **75/78** | **100.00** | **68.83** | **69.23** |
|  | **DT** | **25/27** | **90.20** | **68.63** | **69.23** |
|  |  | **50/52** | **93.46** | **74.51** | **78.85** |
|  |  | **75/78** | **94.77** | **60.78** | **82.69** |
|  | **CNN** | **25/27** | **92.16** | **80.39** | **69.23** |
|  |  | **50/52** | **97.39** | **96.08** | **90.38** |
|  |  | **75/78** | **100.00** | **100.00** | **98.08** |
|  | **Partial Least Squares-Discriminatory Analysis** | **25/27** | **92.81** | **76.47** | **71.15** |
|  |  | **50/52** | **95.42** | **88.24** | **82.69** |
|  |  | **75/78** | **98.04** | **88.24** | **86.54** |
|  | **Stochastic Gradient Descent** | **25/27** | **92.16** | **74.51** | **61.54** |
|  |  | **50/52** | **93.46** | **86.27** | **78.85** |
|  |  | **75/78** | **98.04** | **88.24** | **73.08** |
|  | **Gradient Boosting** | **25/27** | **93.46** | **78.43** | **67.31** |
|  |  | **50/52** | **94.77** | **84.31** | **71.15** |
|  |  | **75/78** | **100.00** | **84.31** | **73.08** |
| **RGB Images** | **LR** | **25/27 ^§^** | **84.97** | **78.43** | **71.15** |
|  |  | **50/52** | **97.39** | **96.08** | **82.69** |
|  |  | **75/78** | **100.00** | **100.00** | **84.62** |
|  | **SVM** | **25/27** | **98.04** | **92.16** | **71.15** |
|  |  | **50/52** | **94.77** | **96.08** | **78.85** |
|  |  | **75/78** | **100.00** | **100.00** | **80.77** |
|  | **NN** | **25/27** | **90.20** | **84.31** | **63.46** |
|  |  | **50/52** | **97.39** | **96.08** | **73.08** |
|  |  | **75/78** | **98.04** | **98.04** | **75.00** |
|  | **DT** | **25/27** | **91.50** | **78.43** | **53.85** |
|  |  | **50/52** | **100.00** | **92.16** | **76.92** |
|  |  | **75/78** | **100.00** | **96.08** | **76.92** |
|  | **CNN** | **25/27** | **94.77** | **78.43** | **71.15** |
|  |  | **50/52** | **97.39** | **96.08** | **82.69** |
|  |  | **75/78** | **100.00** | **100.00** | **84.62** |
|  | **Partial Least Squares-Discriminatory Analysis** | **25/27** | **90.20** | **78.43** | **71.15** |
|  |  | **50/52** | **93.46** | **96.08** | **82.88** |
|  |  | **75/78** | **100.00** | **100.00** | **84.62** |
|  | **Stochastic Gradient Descent** | **25/27** | **91.50** | **78.43** | **63.46** |
|  |  | **50/52** | **95.52** | **92.16** | **78.85** |
|  |  | **75/78** | **100.00** | **100.00** | **80.77** |
|  | **Gradient Boosting** | **25/27** | **92.16** | **88.24** | **67.31** |
|  |  | **50/52** | **98.04** | **90.20** | **78.85** |
|  |  | **75/78** | **98.04** | **98.04** | **79.62** |
| **Hyperspectral images** | **LR** | **25/27 ^§^** | **84.97** | **78.43** | **53.85** |
|  |  | **50/52** | **90.20** | **92.16** | **80.77** |
|  |  | **75/78** | **100.00** | **86.27** | **82.69** |
|  | **SVM** | **25/27** | **88.24** | **76.47** | **50.00** |
|  |  | **50/52** | **98.04** | **96.08** | **78.85** |
|  |  | **75/78** | **100.00** | **96.08** | **84.62** |
|  | **NN** | **25/27** | **90.20** | **74.51** | **51.92** |
|  |  | **50/52** | **93.46** | **76.47** | **78.85** |
|  |  | **75/78** | **90.20** | **84.31** | **78.85** |
|  | **DT** | **25/27** | **91.50** | **76.47** | **51.92** |
|  |  | **50/52** | **97.39** | **78.43** | **76.92** |
|  |  | **75/78** | **100.00** | **84.31** | **80.77** |
|  | **CNN** | **25/27** | **91.50** | **78.43** | **53.85** |
|  |  | **50/52** | **91.50** | **90.20** | **88.46** |
|  |  | **75/78** | **92.81** | **92.16** | **88.46** |
|  | **Partial Least Squares-Discriminatory Analysis** | **25/27** | **92.16** | **78.43** | **48.08** |
|  |  | **50/52** | **95.42** | **90.20** | **76.92** |
|  |  | **75/78** | **100.00** | **90.20** | **78.85** |
|  | **Stochastic Gradient Descent** | **25/27** | **88.89** | **78.43** | **51.92** |
|  |  | **50/52** | **93.46** | **92.16** | **63.46** |
|  |  | **75/78** | **100.00** | **96.08** | **69.23** |
|  | **Gradient Boosting** | **25/27** | **88.24** | **78.43** | **50.00** |
|  |  | **50/52** | **94.77** | **88.24** | **80.77** |
|  |  | **75/78** | **93.46** | **84.31** | **84.62** |

^§^ The number of category samples is expressed as: the number of healthy samples/number of infected samples.
